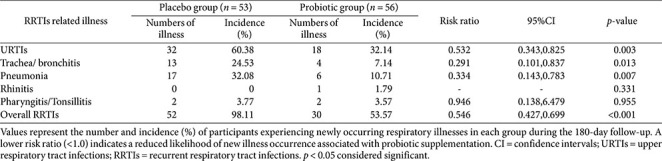# Probiotic Supplementation Reduces RRTIs and Enhances Gut Microbial and Immunity in Children: A Randomized Controlled Trial

**DOI:** 10.4014/jmb.2511.11038

**Published:** 2026-02-03

**Authors:** Ke Chen, Weiwei Ma, Jiayi Zhong, Ping Yang, Nianyang He, Xiaohui Li, Yue Zhai, Jie Yuan, Min-Tze Liong, Changqi Liu, Yuxiu Guan

**Affiliations:** 1 Department of Clinical Nutrition, Chengdu Women’s & Children’s Central Hospital, School of Medicine, University of Electronic Science and Technology of China, P.R. China; 2 College of Pharmacy, Heilongjiang University of Chinese Medicine, P.R. China; 3 Department of Child Health Care, Xindu Maternal and Child Health Care Hospital, P.R. China; 4 Laboratory of Microbiology, Immunology, and Metabolism, DiPROBIO (Shanghai) Co., Limited, Shanghai, P.R. China; 5 School of Exercise and Nutritional Sciences, San Diego State University, P.R. China; 6 Sunflower Pharmaceutical Group Co., Limited, Harbin, P.R. China

**Keywords:** Recurrent respiratory tract infections, Probiotics, Children, Gut-lung axis, *Bifidobacterium*, *Lactiplantibacillus*

## Abstract

Recurrent respiratory tract infections (RRTIs) are a major cause of morbidity in children, and strain-defined probiotics have been proposed as a supportive preventive strategy, although clinical evidence remains limited. In this randomized, double-blind, placebo-controlled trial, 120 children diagnosed with RRTIs received either *Bifidobacterium animalis* subsp. *lactis* XLTG11 and *Lactiplantibacillus plantarum* CCFM8661 or a matched placebo daily for 180 days. The probiotic group demonstrated significantly reduced duration and frequency of fever, cough, upper respiratory tract infections, trachea/bronchitis, pneumonia, and overall RRTI recurrence compared with the placebo group (all *p* < 0.05). Gut microbiota profiling showed clear community differences between groups at day 180, with the probiotic group exhibiting greater abundance of beneficial commensal taxa and the placebo group showing higher representation of opportunistic genera. Functional pathway analysis indicated shifts consistent with enhanced metabolic stability in probiotic recipients. Immune biomarker patterns further supported a more regulated humoral response in the probiotic group, reflected by comparatively stable IgG, IgM, and complement C3 levels over the intervention period. Growth trajectories remained normal in both groups, and no treatment-related adverse events were reported, confirming a favorable safety profile. These findings indicate that long-term supplementation with XLTG11 and CCFM8661 is safe, well tolerated, and effective in reducing RRTI burden in children, while also supporting healthier microbiota and immune patterns. This trial provides evidence for the use of strain-defined probiotics as a complementary approach within pediatric respiratory infection prevention strategies.

## Introduction

Recurrent respiratory tract infections (RRTIs) are common in infants and young children due to physiologic immaturity of the respiratory and immune systems and represent a major clinical and socioeconomic concern worldwide [1]. In China, RRTI diagnosis is based on unusually frequent upper or lower respiratory infection episodes, and affected children often experience repeated symptoms, absenteeism, unnecessary antibiotic use, increased healthcare visits, and substantial family burden. Prevalence varies geographically, approximately 10% in Finland [2], 6% in Italy [3], and up to 20% in China [4], highlighting the need for safe and effective preventive strategies.

The pathogenesis of RRTIs is multifactorial, involving pathogens, environmental exposures, allergy, airway development, epithelial integrity, and immune maturation [5-7]. Immune dysregulation is increasingly recognized as a key contributor; therefore, immunomodulatory approaches have gained interest [1, 3, 6]. However, many current options lack strong pediatric evidence or long-term tolerability.

The gut–lung axis has emerged as a relevant biological framework linking intestinal microbiota to respiratory immune homeostasis [8, 9]. Evidence suggests that targeted microbiota modulation can influence airway inflammation, mucosal immunity, and infection susceptibility. Several trials and meta-analyses report that specific probiotic strains reduce respiratory illness duration and frequency in children [10, 11]. Because probiotic effects are highly strain-specific [12, 13], expert recommendations emphasize strain-level validation when assessing pediatric respiratory benefits [14-16].

*Bifidobacterium animalis* subsp. *lactis* XLTG11 (CGMCC No.18738), isolated from healthy Chinese infants, has demonstrated immune-enhancing and intestinal regulatory functions [17-19]. *Lactiplantibacillus plantarum* CCFM8661 (CGMCC No.5494) modulates gut ecology and cytokine responses in experimental models [20-22]. Although both strains show functional potential, their combined clinical impact on pediatric RRTI recurrence has not been established, underscoring the need for controlled clinical evaluation.

In this randomized, double-blind, placebo-controlled trial in children with RRTIs, we assessed whether daily supplementation with XLTG11 and CCFM8661 improves clinical outcomes, reduces recurrence, and modulates gut microbiota and immune biomarkers. As a strain-defined mechanistic evaluation, the findings cannot be generalized to other probiotic species or commercial mixtures without strain-level evidence.

To our knowledge, this is the first mechanistic, strain-defined randomized trial in Chinese pediatric RRTIs integrating clinical, microbial, and immune data over 180 days. Unlike prior short-term studies focused solely on symptoms, this trial included a secondary outcome of establishing a mechanistic foundation linking microbial metabolic maturation to clinical recovery. By evaluating microbiota composition, KEGG functional maturity, and systemic immune biomarkers over six months, this study advances probiotic research toward a more comprehensive model of pediatric immunity.

## Materials and Methods

### Study Design and Ethical Approval

This randomized, double-blind, parallel-group, placebo-controlled clinical trial was conducted between February and July 2024. Children diagnosed with recurrent respiratory tract infections (RRTIs) were recruited from Chengdu Women’s and Children’s Central Hospital and Xindu Maternal and Child Health Care Hospital. Eligible participants were boys or girls aged 0-14 years who met the 2022 national diagnostic criteria for pediatric RRTIs [23], had not taken other probiotic preparations during the study period, and whose parents or guardians agreed to provide fecal and blood samples. Written informed consent was obtained from all parents or guardians prior to enrollment.

Children were excluded if they were born before 37 gestational weeks, had birth weight <2,500 g or >4,000 g, had used the same probiotic strain within one month prior to enrollment, or had used antibiotics, disinfectants, antifungals, glucocorticoids, or other immunity-modifying medications within 7 days before enrollment. Additional exclusion criteria included malnutrition requiring hospitalization, known hypersensitivity to any ingredients in the study product, moderate or severe anemia, rickets, chronic diarrhea, congenital cardiovascular malformations, severe malnutrition from any cause, congenital or secondary immunodeficiency, nephropathy, cerebral palsy, or long-term immunosuppressive therapy. Children who had taken immune-modulating medications or participated in another clinical study within 3 months prior to enrollment, or were otherwise considered unsuitable by investigators, were excluded.

Participants were withdrawn if they were mis-enrolled or misdiagnosed, if clinical evaluation data were unavailable, if allergic reactions occurred, if the product could not be retained in the gastrointestinal tract, or if clinical deterioration required transfer to the Pediatric Intensive Care Unit (PICU). The study was approved by the Ethical Committee of Chengdu Women’s and Children’s Central Hospital (IEC-C-007-V.02), conducted in accordance with the Declaration of Helsinki, and registered at the Chinese Clinical Trial Registration Center (ChiCTR2300077434). All primary (RRTI symptom frequency and severity) and secondary outcomes (gut microbiota composition and immune biomarkers) reported in this manuscript were prespecified in the registered protocol, and no outcomes were added, modified, or omitted from the preregistered study plan.

### Randomization and Blinding

Randomization was performed using block randomization with a fixed block size of four to ensure balanced assignment across the 180-day enrollment period. An independent biostatistician generated the allocation sequence using a computer-based random number generator and stratification was not applied. Allocation concealment was maintained through a centralized procedure, where the randomization list was transmitted only to the product manufacturer, who packaged the probiotic and placebo sachets in identical, sequentially coded containers. Investigators, caregivers, participants, laboratory staff, data managers, and statisticians were fully blinded to group allocation throughout the trial, and the randomization code was not broken until all clinical and microbiome analyses were completed.

Sample size estimation was based on prior RRTI data where treatment efficacy was 92% (47/51) in the probiotic arm versus 74% (37/50) in placebo [24]. Assuming α = 0.05, u = 2.799, the required total enrollment was 100 children. To ensure adequate statistical power and an anticipated dropout rate of 20%, 120 children (60 per group) were enrolled.

### Intervention

Participants in the probiotic group received one oral sachet daily containing maltodextrin (50%) with a combined total dose of 1 × 10^10^ CFU per sachet of *B. animalis* subsp. *lactis* XLTG11 and *L. plantarum* CCFM8661. The sachet could be taken directly or dissolved in liquids at < 45°C. Placebo group participants received identical sachets containing maltodextrin only. Daily supplementation continued for 180 days. If vomiting occurred within 30 min after ingestion, a second sachet was administered (maximum once within 4 h). All dosing and re-dosing events were recorded in case report forms (CRFs). Standard pediatric care continued as clinically indicated. If concurrent antibiotic therapy was required, probiotic/placebo intake was separated by a minimum of 3 h.

### Data Collection

After enrollment, clinical data were collected using standardized CRFs. The primary outcome was the frequency of RRTI recurrence during the 180-days study period. Secondary endpoints included fecal microbiota composition (16S rRNA sequencing), taxonomic profiling and predicted functional pathways, and plasma immune biomarkers (IgA, IgG, IgM, C3, C4) at baseline and after 180 days. Clinicians recorded symptom duration, medication use, and clinical manifestations including fever, cough, nasal congestion, rhinorrhea, and pharyngeal congestion.

### Sample Collection

Fecal samples were collected at baseline and after 180 days of intervention into RNAlater tubes and stored at -80°C. Peripheral fasting blood samples (1-2 ml) were collected, centrifuged at 3,000 r/min for 10 min, and plasma stored at 2-4°C. Plasma IgA, IgG, IgM, C3, and C4 concentrations were quantified using commercial ELISA kits (Shanghai Enzyme Linked Biotechnology Co., Ltd., China).

### DNA Extraction and 16S rRNA Gene Sequencing

Genomic DNA was extracted using the QIAamp Fast DNA Fecal Mini Kit (Qiagen, USA). The V3-V4 region of the bacterial 16S rRNA gene was amplified using primers 341F/806R with the TransGen AP221-02 kit and sequenced on the Illumina MiSeq platform using paired-end 2 × 300 bp reads. Raw fastq files underwent quality control using fastp, including adapter trimming, removal of bases below Q30, and exclusion of reads shorter than 50 bp. High-quality paired-end reads were merged and screened for chimeras before downstream processing. Operational taxonomic units (OTUs) were clustered at 97% similarity using Uparse v7.0.1001 and QIIME 1.9.1, and taxonomy was assigned using the RDP classifier. Alpha diversity (Observed species, Shannon, Pielou_J, PD_FAITH) and beta diversity (Bray–Curtis, weighted and unweighted UniFrac) indices were calculated using the normalized OTU table. Intergroup differential abundance was assessed using the Kruskal–Wallis test. All subsequent ecological and functional analyses were performed on the filtered and normalized OTU table.

### Rare-Taxa Filtering and Normalization

After initial quality control, chimera removal and taxonomic assignment, sequences annotated as chloroplasts and mitochondria, and singleton OTUs (total count = 1) were removed. To reduce spurious signals from very-low-prevalence/low-abundance taxa prior to diversity and differential-abundance testing, the OTU table was filtered using both prevalence and mean-abundance criteria: OTUs were retained only if present in ≥5% of samples (prevalence) and with mean relative abundance across samples ≥0.01%. These thresholds were chosen to remove sample-specific sequencing noise while retaining low-abundance taxa that are consistent across subjects. After filtering, counts were normalized for library size using cumulative sum scaling (CSS) for community analyses and variance-stabilizing size factors (DESeq2) for model-based differential tests.

### Differential Taxonomy and Pathway Analysis

LEfSe analyses (α = 0.05; LDA ≥ 2.0) were performed on the prevalence- and abundance-filtered, normalized OTU table to evaluate within-group longitudinal changes (baseline vs day 180). In accordance with the pre-specified statistical analysis plan and the exploratory nature of the microbiome outcomes, no between-group differential abundance tests were conducted, as the study was not powered to detect between-group microbial differences. Functional prediction of metabolic pathways was performed using PICRUSt2 based on 16S data, and annotated to KEGG pathways. Statistical comparisons for functional enrichment outputs were conducted using STAMP v2.1. Data harmonization and visualization were performed using Gi-MAPS.

### Statistical Analysis

Primary analyses followed intention-to-treat (ITT) and per-protocol (PP) principles. Normality was tested using the Kolmogorov–Smirnov test. Continuous variables were expressed as mean ± standard deviation or median (P25, P75), and categorical variables as number (percentage). Log transformation was applied where appropriate. Student’s t-test was used for normally distributed data; the Mann–Whitney U test for non-normally distributed data; and χ^2^ tests for categorical data. Negative binomial regression estimated relative risk and 95% confidence intervals for symptom duration outcomes. All analyses were conducted using IBM SPSS Statistics 29.0.2 (Mac).

### Transparency Statement

To promote transparency and reproducibility, the complete analysis pipeline, anonymized data structure, and code metadata are documented in the supplementary materials and can be accessed upon reasonable request. All laboratory and computational protocols followed standardized procedures to ensure methodological consistency and reproducibility.

## Results

### Basic Clinical and Demographic Characteristics

**Fig. 1:**
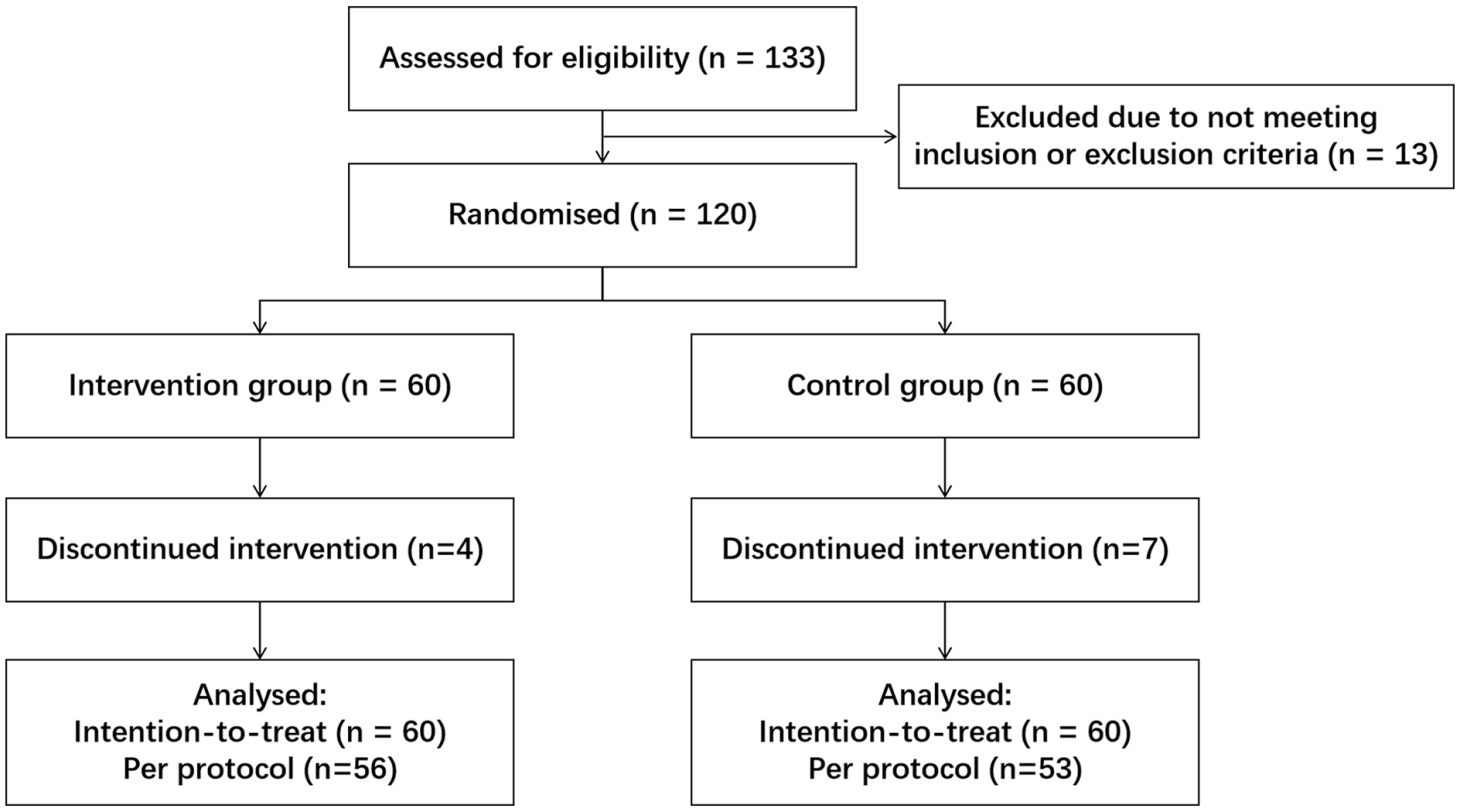
CONSORT flow diagram of study enrollment, allocation, follow-up, and analysis. Diagram illustrating screening, randomization, intervention allocation, follow-up, and dataset inclusion for analysis. Four participants in the probiotic group and seven in the placebo group dropout due to being uncontactable. Analyses were conducted using a per-protocol (PP) approach, based on the sample sizes after these dropouts.

All 120 eligible children with RRTIs were enrolled, randomized, and allocated into the probiotic or placebo group (*n* = 60 per group). During the 180-day intervention, 4 participants in the probiotic group and 7 in the placebo group discontinued participation due to loss to follow-up (uncontactable), as shown in the CONSORT diagram (Fig. 1). Because no clinical or microbiota endpoint data were obtained from these individuals, all efficacy and microbiota analyses were conducted under a per-protocol (PP) framework, using the sample sizes after dropout, (*n* = 109, *n* = 56 probiotic, *n* = 53 placebo). All analyses were performed using PP dataset. No adverse events attributable to the study product were observed.

Baseline demographic and clinical characteristics did not differ significantly between groups, including age, sex, gestational age, parental education level, household income, household residence type, mode of delivery, household size, or history of allergic disease (all *p* > 0.05; Table 1). Throughout the study, no gastrointestinal or systemic adverse reactions (including abdominal pain, nausea, vomiting, diarrhea, constipation, appetite change, fever, or allergic symptoms) were reported.

### Burden of Respiratory Symptoms and RRTI-Related Illnesses

Across the full observation period, the probiotic group experienced significantly shorter total durations of fever, cough, nasal congestion, pharyngeal hyperemia, URTIs, trachea/bronchitis, pneumonia, and overall total RRTIs compared with the placebo group (all *p* < 0.05; Table 2). Following adjustment for multiple demographic covariates (sex, age months, gestational age, parental education, household income, residence type, mode of delivery, household population), negative binomial regression demonstrated significantly reduced risk associated with probiotic supplementation for nasal congestion, runny nose, pharyngeal hyperemia, URTIs, trachea/bronchitis, pneumonia, otitis media, rhinitis, pharyngitis/tonsillitis, and overall total RRTIs (all *p* < 0.05; Table 3).

### Incidence of Newly Occurring RRTI Episodes

The incidence of newly occurring RRTI-related illnesses was consistently lower in the probiotic group than the placebo group during follow-up (Table 4). Compared with the placebo group, the probiotic group demonstrated significantly reduced risk of new URTIs (32.1% vs 60.4%, RR = 0.532; 95% CI: 0.343, 0.825), trachea/bronchitis (7.1% vs 24.5%, RR = 0.291; 95% CI: 0.101, 0.837), pneumonia (10.7% vs 32.1%, RR = 0.334, 95% CI: 0.143, 0.783) and overall RRTIs (53.6% vs 98.1%, RR = 0.546; 95% CI: 0.427, 0.699) (all *p* < 0.05).

### Effect of Probiotic Intervention on Growth Parameters

Weight, length/height, and head circumference were comparable between groups at baseline (all *p* > 0.05). All indices increased over 180 days in both groups, consistent with normal growth, and remained similar between groups (*p* > 0.05), indicating that XLTG11+CCFM8661 supported healthy physical development.

### Effect of Probiotic Intervention on Fecal Gut Microbiota

**Fig. 2:**
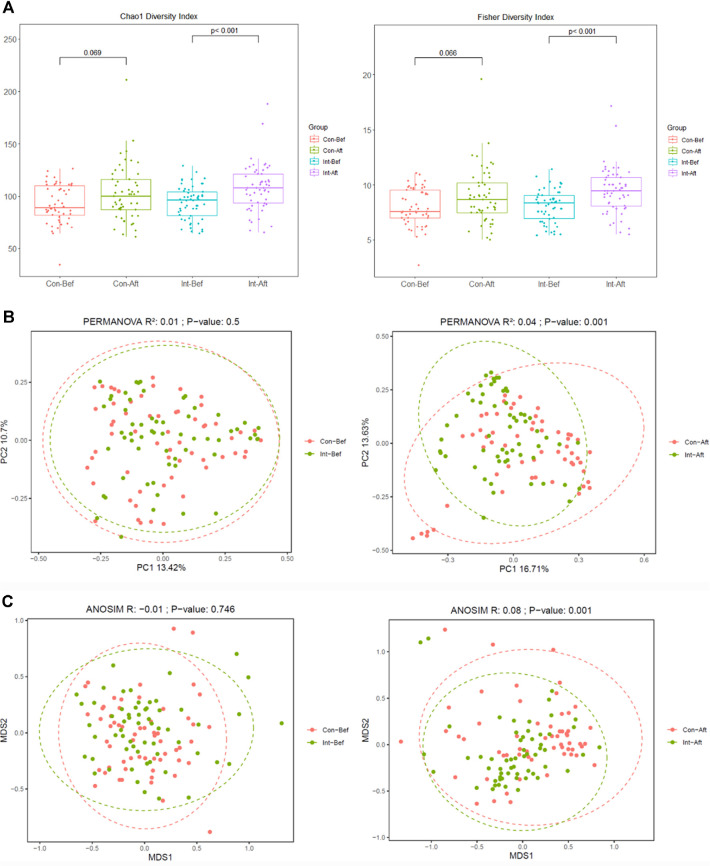
Gut microbiota diversity before and after probiotic intervention. Changes in alpha diversity indices (**A**) including Chao1 and Fisher across baseline and day-180 samples in the probiotic and placebo groups. Boxes represent interquartile ranges, horizontal lines indicate medians, and dots represent individual samples. Beta diversity analyses based on Bray-Curtis dissimilarity assessed by PCoA (**B**) and NMDS (**C**). Ellipses represent 95% confidence intervals for group clustering. PCoA = Principal Coordinates Analysis; NMDS = Non-metric Multidimensional Scaling. Significant at *p* <0.05. Pla-Bef = Placebo group baseline; Pla-Aft = Placebo group day-180; Pro-Bef = Probiotic group baseline; Pro-Aft = Probiotic group day-180.

**Alpha and beta diversity.** After 180 days of probiotic supplementation, alpha diversity, as measured by the Chao1 and Fisher indices, showed a significant increase in the probiotic group, indicating enhanced microbial richness and evenness within individuals. This improvement was not observed in the placebo group (Fig. 2A), suggesting that probiotic intake promoted a more diverse and potentially resilient gut ecosystem. In parallel, beta diversity analysis based on Bray–Curtis dissimilarity revealed no significant differences between groups at baseline (Fig. 2B and 2C). However, after 180 days, clear separation between the probiotic and placebo groups emerged, reflecting distinct microbial community compositions associated with probiotic use. Notably, these microbiome changes coincided with a reduction in recurrent respiratory tract infection (RRTI) symptom burden in the probiotic group, underscoring the potential clinical relevance of enhanced gut microbial diversity in supporting host immunity over time.

**Fig. 3:**
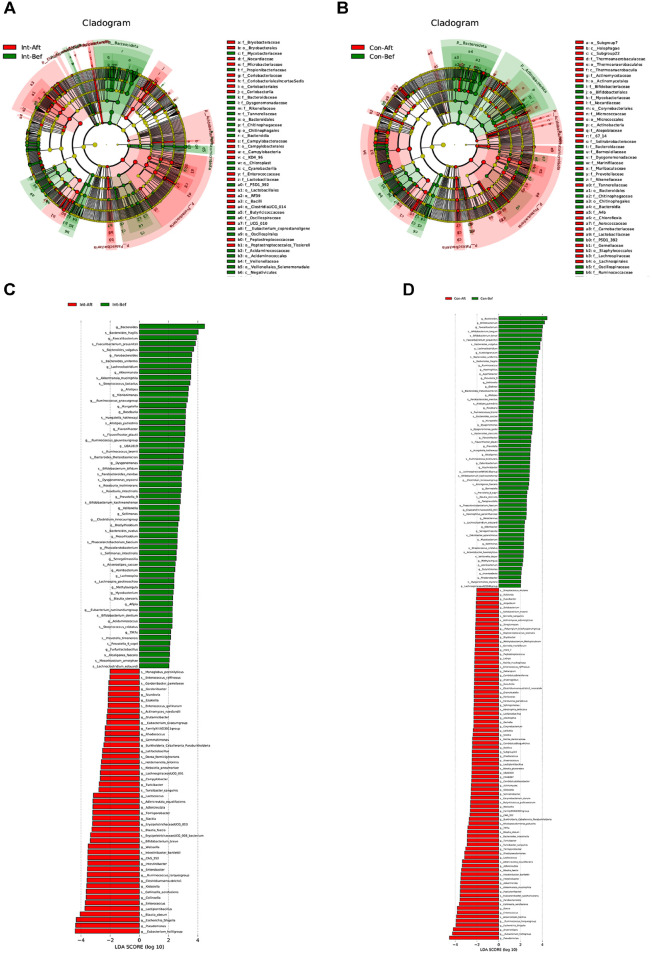
LEfSe-based identification of differentially abundant taxa before and after the intervention within each study group. Analyses were performed on the prevalence- and abundance-filtered, normalized OTU table (OTUs retained only if present in ≥5% of samples and with mean relative abundance ≥0.01%). LEfSe parameters: α = 0.05 and LDA ≥ 2.0. Because the study was not powered for between-group microbial comparisons and the statistical analysis plan specified within-participant analyses, Figure 3 displays only changes within group; thus, the results characterize longitudinal microbial shifts but do not represent probiotic-specific effects. Cladograms and LDA bar plots highlight taxa enriched in the probiotic group (**A, C**) and the placebo group (**B, D**) after the 180-day period. LDA = linear discriminant analysis; LEfSe = Linear Discriminant Analysis Effect Size. Pla-Bef = Placebo group baseline; Pla-Aft = Placebo group day-180; Pro-Bef = Probiotic group baseline; Pro-Aft = Probiotic group day-180.

**Identification of biomarkers and discriminatory taxa.** LEfSe-based analysis revealed distinct microbial remodeling trajectories between the probiotic and placebo groups over the 180-day intervention period (Fig. 3). Children receiving probiotic supplementation showed shifts toward fermentative and immunometabolic taxa, whereas the placebo group transitioned toward aerotolerant, pro-inflammatory, and opportunistic signatures, consistent with the differing clinical symptom profiles observed between groups.

In the probiotic group, enrichment centered on SCFA-producing and mucosa-associated commensals. By day 180, the *Eubacterium hallii* group, *Eubacterium siraeum* group, *Eubacterium ruminantium* group, *Faecalibacterium*, *Adlercreutzia*, *Catenibacillus*, and *Collinsella* increased. At the species level, elevations in *Eubacterium rectale*, *Eubacterium eligens*, and related Clostridia XIVa/IV taxa indicated enhanced butyrate-linked metabolic potential, while *Adlercreutzia equolifaciens* and *Collinsella aerofaciens* supported polyphenol and immunometabolic pathways. *Akkermansia muciniphila* was detected with a higher effect size at baseline compared to day 180 in the probiotic group, whereas this temporal shift was not observed in the placebo group. While this pattern represents a within-group change over the intervention period, it may reflect a shift in mucosa-associated taxa consistent with a stable mucus–microbiota relationship, although no causal interpretation can be drawn within the limits of the present analysis.

Additional probiotic-associated changes included expansion of classical cross-feeding fermenters such as *Gemmiger formicilis*, *Anaerostipes hadrus*, and *Coprococcus comes*. The appearance of *Fusicatenibacter saccharivorans* and *Gordonibacter pamelaeae* at low but consistent abundance suggests maturing capacities for polyphenol transformation and secondary metabolite generation. Together, these patterns reflect a shift toward an anaerobic, SCFA-oriented community structure.

In contrast, the placebo group exhibited increased prevalence of facultative and opportunistic taxa. By day 180, *Escherichia–Shigella*, *Enterobacter*, *Burkholderia*, *Ralstonia*, *Raoultella*, *Pseudomonas*, and *Campylobacter* had emerged, forming an aerotolerant consortium associated with inflammatory or unstable gut environments in children. The rise of *Enterococcus faecalis* and *Staphylococcus haemolyticus* further suggested susceptibility to opportunistic colonization. Environmental genera such as *Sphingomonas*, *Rhodopseudomonas*, and *Methylobacterium–Methylorubrum* also appeared, indicating a pattern consistent with oxidative or disturbed microbial states. Meanwhile, beneficial butyrate producers such as *Faecalibacterium prausnitzii* and the *Eubacterium hallii* group did not expand, suggesting limited functional maturation in the placebo microbiota.

Overall, these divergent patterns indicate that probiotic supplementation favored a coherent fermentative microbiota, whereas the placebo group trended toward an aerotolerant, opportunist-enriched configuration. *Lactiplantibacillus* was detected as a significant taxon in both groups after the 180-day period, with a higher LDA score in the probiotic group than placebo group (LDA score 3.73 vs. 2.48). As the study was not powered for between-group comparisons, this represents an observation rather than a confirmed treatment effect.

Taken together, these findings suggest that XLTG11-directed intervention supported a stable, SCFA-enriched microbial environment while limiting expansion of pathobionts, providing a mechanistic context for the clinical improvements observed and highlighting the role of ecological stabilization, rather than increased diversity alone, in early childhood probiotic efficacy.

### Functional Pathway Enrichment Analysis

**Fig. 4:**
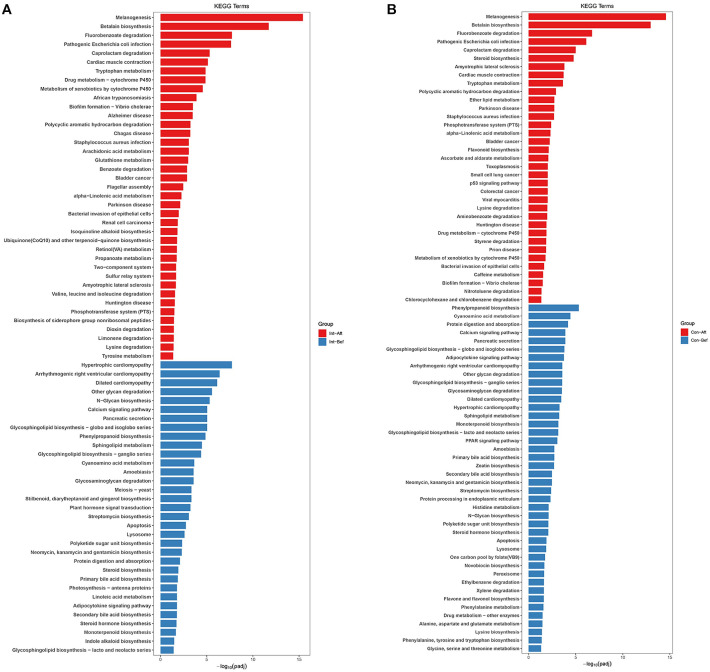
Differential KEGG pathway enrichment in the gut microbiome of participants receiving (A) probiotic group or (B) placebo group. Significantly enriched pathways over time (*p* < 0.05) for each group are shown. Statistical comparisons for functional enrichment outputs were conducted using STAMP v2.1 (*p* < 0.05). KEGG = Kyoto Encyclopedia of Genes and Genomes. Pla-Bef = Placebo group baseline; Pla-Aft = Placebo group day-180; Pro-Bef = Probiotic group baseline; Pro-Aft = Probiotic group day-180.

KEGG pathway analysis identified significant within-group functional shifts from baseline to day 180 in both the probiotic and placebo groups (Fig. 4). To avoid selective reporting, all enriched pathways were examined across major functional domains. As the study was not powered for between-group comparisons, findings reflect longitudinal changes within each group only.

In the probiotic group, post-intervention enrichment spanned multiple metabolic categories. Amino-acid pathways (tryptophan, lysine, valine/leucine/isoleucine, tyrosine, glutathione), lipid pathways (arachidonic acid, α-linolenic acid), and vitamin/cofactor pathways (ubiquinone/CoQ10, retinol) were consistently represented, along with shifts in carbohydrate metabolism (propanoate metabolism) and regulatory modules (two-component system). Numerous xenobiotic degradation pathways (fluorobenzoate, caprolactam, PAH, dioxin) and several disease-interaction pathways (pathogenic *E. coli*, *S. aureus*, African trypanosomiasis, Chagas disease) were also enriched, reflecting broad functional remodeling rather than treatment-specific signatures. Overall, the probiotic group showed a more concentrated pattern of enrichment in core metabolic functions, particularly amino-acid, lipid, and cofactor metabolism.

In the placebo group, enriched pathways also covered diverse domains, including xenobiotic degradation (fluorobenzoate, aminobenzoate, styrene, nitrotoluene, P450-related metabolism), lipid metabolism (steroid biosynthesis, ether lipids, α-linolenic acid), and several amino-acid pathways. The placebo profile was more dispersed, with substantial enrichment of glycan pathways (multiple glycosphingolipid series, N-glycan biosynthesis, glycosaminoglycan degradation), bile-acid metabolism (primary and secondary bile acids), and multiple cardiovascular disease pathways. Infection-related modules (pathogenic *E. coli*, *S. aureus*, Toxoplasmosis) also appeared, as in the probiotic group.

Overall, both groups exhibited broad and multidirectional remodeling across metabolic and disease-related pathways. Many core functions, including carbohydrate and amino-acid metabolism, xenobiotic degradation, and cofactor biosynthesis, were enriched in both groups, consistent with general temporal maturation rather than intervention-specific effects. Although amino-acid, lipid, and cofactor pathways appeared more frequently in the probiotic group, similar categories were present in the placebo group. Conversely, the placebo group displayed more heterogeneous enrichment dominated by glycan and bile-acid metabolism. These findings highlight that KEGG outputs reflect community-level remodeling within each group and should be interpreted as broad functional patterns rather than isolated pathway differences.

### Effect of Probiotic Intervention on Immune Parameters

**Fig. 5:**
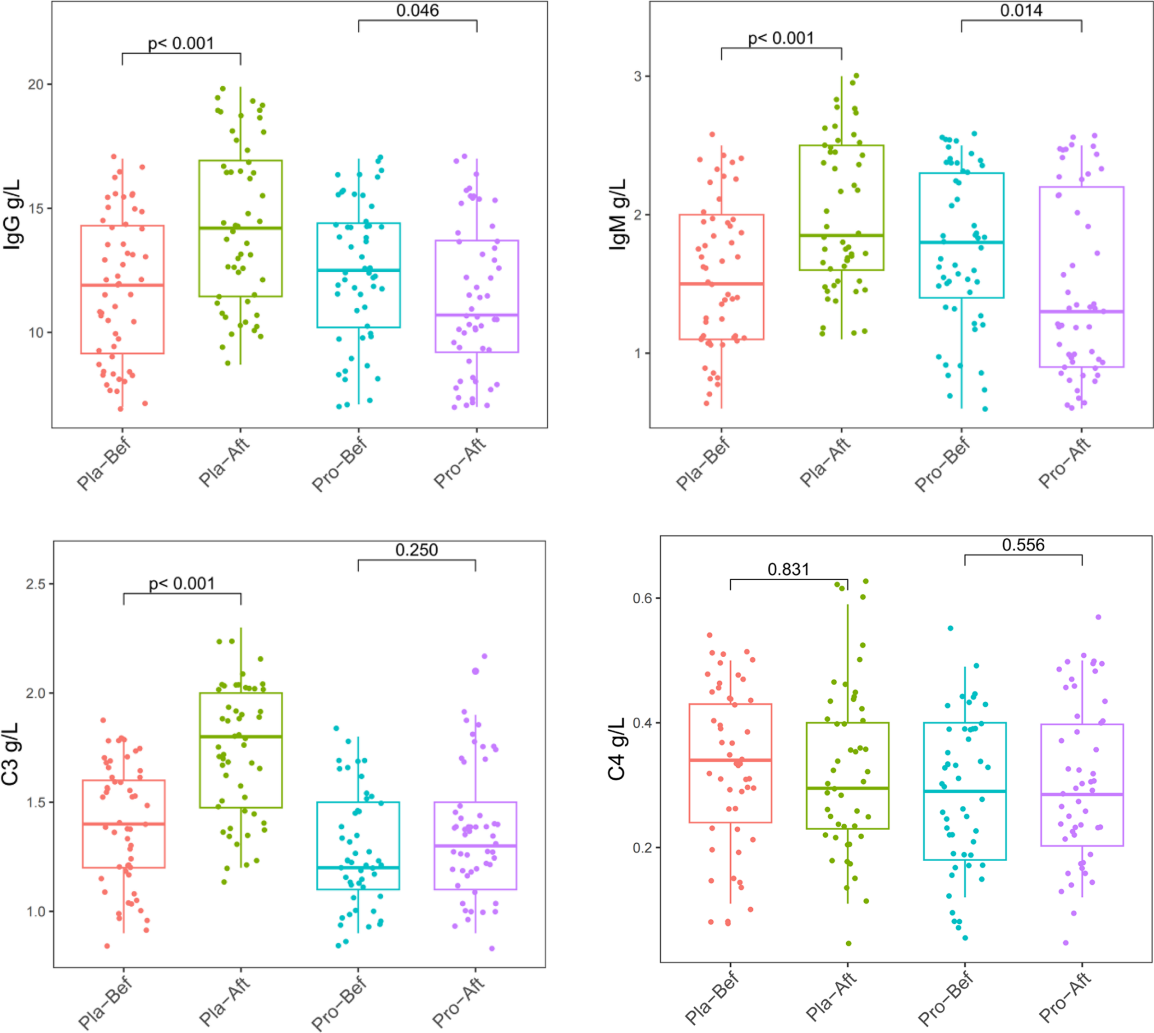
Plasma immune biomarker concentrations before and after intervention. Changes in plasma immunoglobulin-G (IgG), immunoglobulin-M (IgM), Complement C3, and Complement C4 between baseline and day-180 in the probiotic and placebo groups. Pla-Bef = Placebo group baseline; Pla-Aft = Placebo group day-180; Pro-Bef = Probiotic group baseline; Pro-Aft = Probiotic group day-180. *p* < 0.05 considered significant.

The probiotic group exhibited a distinct profile of immune homeostasis compared to the placebo group (Fig. 5). Significant increases in IgG, IgM, and Complement C3 in the placebo group (*p* < 0.05) suggested immune activation, whereas these parameters remained stable or decreased in the probiotic group, indicating a more regulated humoral immune response. Complement C4 remained unchanged in both groups.

## Discussion

### Importance of the Current Findings

The central contribution of this study is the demonstration that a 180-day probiotic intervention altered gut microbial community structure and taxonomic composition in ways that aligned with improved clinical outcomes. LEfSe identified the taxa most responsible for divergences between groups, with the probiotic arm showing enrichment of anaerobic, SCFA-producing and cross-feeding taxa (*Eubacterium* groups, *Faecalibacterium*, *Anaerostipes*, *Coprococcus*, *Gemmiger*, *Adlercreutzia*, *Akkermansia*), while the placebo group showed increases in aerotolerant or opportunistic taxa (*Escherichia–Shigella*, *Enterobacter*, *Enterococcus*, *Pseudomonas*, *Burkholderia*, *Ralstonia*). These taxonomic shifts provide insight into the ecological processes accompanying clinical improvements[25].

KEGG analysis indicated broad, multidirectional functional remodeling within both groups over time. Enriched pathways spanned amino-acid, lipid, carbohydrate, and xenobiotic metabolism, as well as disease-interaction modules, without exclusive patterns specific to either the probiotic or placebo group. Rather than indicating distinct mechanistic routes, these changes reflected generalized functional adaptation of the gut ecosystem. Integrating LEfSe with KEGG outputs therefore enabled inference of functional tendencies associated with observed taxonomic shifts while avoiding overinterpretation of individual pathway modules[26, 27].

These microbial features coincided temporally with clinical improvements in the probiotic group, including reductions in fever, cough, URTI episodes, bronchitis/tracheitis, and total RRTIs, providing ecological context for symptom reduction[28-30]. The enrichment of SCFA-associated taxa and maintenance of mucosa-linked genera such as Akkermansia offer plausible mechanistic anchors, but functional pathways should be interpreted as part of broad community remodeling rather than as group-specific signatures.

*Lactiplantibacillus* was detected in both groups after 180 days, with a higher LDA score in the probiotic arm. This pattern should be interpreted cautiously, as pediatric microbiomes are dynamic and 16S sequencing cannot distinguish the administered strain from endogenous species[31]. Furthermore, the study was not powered for between-group microbial comparisons; thus, the presence of *Lactiplantibacillus* represents an observational finding rather than a probiotic-specific effect. Future studies incorporating strain-targeted assays or shotgun metagenomics will be needed to confirm true engraftment.

### Comparison with Existing Literature and Current Clinical Practice

Current clinical management of pediatric RRTIs centers on supportive care (antipyretics, hydration), targeted antibiotics for confirmed bacterial illness, and preventive strategies such as vaccination, hygiene, and exposure reduction [32]. For children with frequent recurrences, clinicians may consider prophylactic measures, environmental modification, or immunological evaluation [33]. Microbiome-directed therapies, including probiotics and related approaches, show growing interest but remain heterogeneous in evidence due to short follow-up, non-standardized strains or doses, and limited functional microbiome data[34, 35].

Our study differs from prior work in several ways that enhance translational relevance. First, the 180-day intervention enabled evaluation of sustained ecological change rather than transient colonization. Second, the use of strain-defined probiotics combined with integrated microbiome analyses (diversity metrics, LEfSe biomarkers, KEGG functions) supports mechanistic interpretation, a gap in many probiotic trials[25, 27]. Third, clinical endpoints were comprehensive and corroborated by immune markers, improving internal consistency. Fourth, the study showed excellent safety and high retention in the per-protocol cohort, supporting feasibility and generalizability.

Collectively, these features position the trial not only as an efficacy assessment but also as a mechanistic demonstration that microbiome ecological repair, via taxonomic and functional maturation, can translate into meaningful reductions in RRTI burden. This integrated approach extends beyond symptom-only studies by linking microbial taxa and metabolic capacity to clinical outcomes, offering a framework for rational microbiome-based strategies in pediatric respiratory prevention.

### Mechanistic Considerations

Mechanistically, the clinical improvements associated with probiotic supplementation may relate to taxonomic features observed in the intervention arm rather than specific KEGG pathways, which showed broad overlap between groups. The expansion of SCFA-producing and cross-feeding taxa, such as *Eubacterium hallii*, *Faecalibacterium prausnitzii*, *Coprococcus comes*, and *Anaerostipes hadrus*, supports increased potential for butyrate and propionate production, metabolites known to promote epithelial energy supply, regulatory T-cell induction, and attenuation of inflammatory cytokines [36, 37].

Although KEGG pathways did not differ exclusively between groups, several modules associated with amino-acid, lipid, and cofactor metabolism appeared more frequently in the probiotic group, consistent with functional maturation of the microbial community. These tendencies align with an immune profile characterized by more stable IgG/IgM levels and reduced complement activation relative to placebo, patterns compatible with SCFA-linked immune regulation [38, 39].

Together, these observations suggest that probiotic-driven taxonomic maturation, rather than specific KEGG pathway exclusivity, may have contributed to enhanced ecological stability and reduced RRTI symptom burden.

### Limitations

This study has several considerations that, while methodological limitations, also reflect its rigorous design. First, although amplicon sequencing with KEGG-inferred functions provides lower gene-level resolution than shotgun metagenomics or metabolomics, it enabled cost-effective, high-throughput profiling in a large pediatric cohort. Integrating taxonomic data with LEfSe and KEGG analyses strengthened biological interpretation by yielding coherent taxonomic–functional insights.

Second, the study population represented a specific pediatric cohort with relatively uniform environmental and socio-economic backgrounds, which may limit generalizability. However, this controlled context improved internal validity and allowed precise assessment of probiotic efficacy in a high-incidence RRTI setting, supporting future multicenter validation.

Third, direct metabolite quantification (*e.g.*, SCFAs, equol, urolithins) was not performed. Nevertheless, the concordance between SCFA-producing taxa (LEfSe) and SCFA-associated pathways (KEGG) provides biologically consistent support for inferred metabolic activity, consistent with recommended analytical triangulation in microbiome trials [40]. Ongoing follow-up studies include fecal and plasma metabolomics to directly link microbial function with immune markers.

Use of a per-protocol dataset may overestimate effect sizes compared with ITT analyses, but it ensured data integrity by focusing on complete, adherent datasets [41]. High retention and absence of treatment-related adverse events reinforce the robustness of the findings.

The microbiome analyses were exploratory secondary outcomes, and the trial was not powered to detect between-group microbial differences. Following the pre-specified analysis plan, differential abundance testing was limited to within-participant comparisons (baseline vs. day 180), and these changes should not be interpreted as probiotic-specific effects. Larger studies with stratified randomization and longitudinal mixed-effects modeling will be needed to test true between-group microbial differences. Additionally, the 16S rRNA V3–V4 approach limits strain-level resolution and detection of ultra-rare taxa; strain-targeted qPCR or shotgun metagenomics would more definitively characterize probiotic-specific and low-abundance microbial dynamics.

Finally, while the 180-day window captures medium-term outcomes, it does not address longer-term sustainability post-supplementation. The longitudinal design implemented here provides a baseline for planned follow-up to assess durability and timing of probiotic effects.

Overall, despite these limitations, the study’s integrated clinical–microbiome design supports scientifically robust, translationally meaningful conclusions and provides a strong foundation for future clinical application and communication.

### Future Outlook and Clinical Implications

These findings highlight the therapeutic potential of microbiota-directed interventions for preventing or mitigating RRTIs in children, particularly during critical windows of immune development. The consistent alignment of clinical improvement with microbial maturation and functional stabilization underscores the role of probiotics as ecological modulators rather than transient colonizers.

Long-term, such interventions may contribute to reduced antibiotic exposure, improved immune homeostasis, and lower incidence of secondary atopic or inflammatory conditions [42]. Future clinical programs should explore personalized probiotic formulations tailored to baseline microbiota profiles and host immune phenotypes, potentially supported by functional metabolomic biomarkers.

### Integrative Microbiome–Immune Axis Interpretation

Integrating taxonomic biomarkers with functional pathway tendencies provides an ecologically grounded model of the pediatric gut–lung axis. LEfSe demonstrated enrichment of butyrogenic and cross-feeding taxa (*Eubacterium* groups, *Faecalibacterium*, *Anaerostipes*, *Coprococcus*, *Gemmiger*), as well as *Akkermansia* and *Adlercreutzia*, in the probiotic group. These organisms support butyrate synthesis, mucin-associated barrier maintenance, and polyphenol conversion into metabolites with immunomodulatory roles [42, 43].

KEGG results, although not group-specific, showed that both groups exhibited shifts across amino-acid, lipid, carbohydrate, and xenobiotic pathways. The somewhat more frequent appearance of amino-acid and cofactor biosynthesis modules in the probiotic arm is consistent with the metabolic capacities expected from the enriched SCFA-producing taxa [44–46].

These microbiome features align with immune measurements indicating more restrained humoral activation in the probiotic group (stable IgG/IgM and C3 vs. increases in placebo) and correspond to reduced respiratory symptom frequency and duration [47-50]. Collectively, these data support a plausible ecological–immune pathway: probiotic-associated taxonomic maturation promotes metabolic stability, which in turn contributes to improved mucosal and systemic immune balance in children with RRTIs [51].

## Conclusion

This study shows that 180-day supplementation with *B. animalis* subsp. *lactis* XLTG11 and *L. plantarum* CCFM8661 in children with RRTIs reduces respiratory symptom burden and shifts the gut microbiota toward a more resilient, functionally mature profile. Integrated analyses of diversity, SCFA-linked taxa, and immune markers provide a mechanistic basis for how probiotic modulation can enhance host resilience. These results lay a solid foundation for preventive, microbiota-guided approaches in pediatric infectious and immune health. The findings support the potential incorporation of probiotic-based microbiome modulation into pediatric preventive care, particularly in the context of antibiotic stewardship and non-pharmacologic management. The demonstrated gut–lung axis framework offers a translational rationale for including microbiome-directed strategies within public health prevention models.

## Figures and Tables

**Table 1:** Baseline clinical and demographic characteristics of participants.

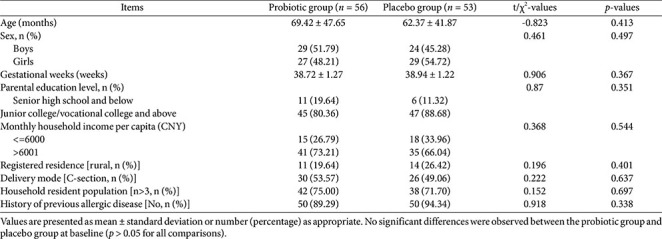

**Table 2:** Burden of respiratory symptoms and RRTIs-related illness during the study period.

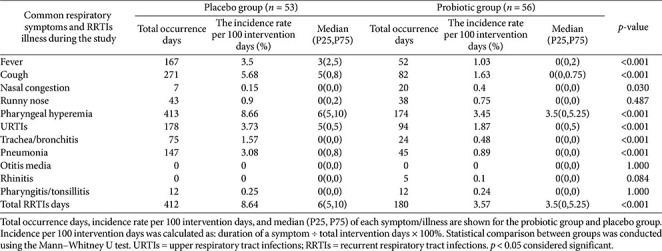

**Table 3:** Adjusted effects of probiotic intervention on respiratory symptoms and RRTIs-related illness derived from negative binomial regression.

Common respiratory symptoms and RRTIs illness during the study	B (Regression coefficient)	95% CI	*p*-value
Fever	-0.021	(-0.230, 0.188)	0.844
Cough	-0.063	(-0.250, 0.114)	0.483
Nasal congestion	-0.551	(-0.803, -0.300)	<0.001
Runny nose	-0.599	(-0.802, -0.397)	<0.001
Pharyngeal hyperemia	-0.977	(-1.263, -0.692)	<0.001
URTIs	-0.639	(-0.909, -0.369)	<0.001
Trachea/bronchitis	-0.859	(-1.121, -0.597)	<0.001
Pneumonia	-0.821	(-1.113, -0.529)	<0.001
Otitis media	-1.006	(-1.276, -0.736)	<0.001
Rhinitis	-1.263	(-1.593, -0.934)	<0.001
Pharyngitis/tonsillitis	-0.763	(-1.180, -0.346)	<0.001
Total RRTIs days	-0.790	(-1.103, -0.477)	<0.001

Negative binomial regression was performed adjusting for multiple demographic covariates including sex, age (months), gestational age, parental education, household income, residence type, mode of delivery, and household population. A negative regression coefficient (B) indicates a reduced risk of symptom occurrence associated with probiotic group and placebo group. URTIs = upper respiratory tract infections; RRTIs = recurrent respiratory tract infections. *p* < 0.05 considered significant.

**Table 4:** Morbidity of newly occurring RRTIs-related illnesses during the study period.